# Secondhand Tobacco Smoke Exposure and Associated Factors among College Students on Campus and in the Home: A Preliminary Study

**DOI:** 10.3390/ijerph9010212

**Published:** 2012-01-16

**Authors:** Eun-Kyung Kim, Jina Choo

**Affiliations:** 1 Department of Nursing, Suwon Science College, San 9-10, Botong-ri, Jeongnam-myun, Hwaseong City, Geonggi-do 445-742, Korea; Email: kek91@lycos.co.kr; 2 College of Nursing, Korea University, Anam-dong, Seongbuk-gu, Seoul 136-705, Korea

**Keywords:** secondhand smoke, tobacco, environmental health, Korea

## Abstract

To explore the prevalence of secondhand tobacco smoke (SHS) exposure of college students at two locations, *i.e.*, on campus and in the home, and to identify factors associated with SHS exposure at each location, a preliminary cross-sectional study was conducted on 1754 nonsmoking students from two universities in Korea. In total, 83.1% were exposed to SHS at least once a week on campus or at home; the average SHS exposure was 3.4 times per week. Specifically, 79.7% and 23.5% were exposed to SHS on campus and in the home, respectively. On campus, SHS exposure was significantly more prevalent in freshmen and sophomore students. In the home, SHS exposure was significantly more prevalent among females, those with smokers in their families, and those who rated their health as poor. SHS exposure was common among nonsmoking college students, with more than two-thirds exposed on campus. The prevalence of SHS exposure was greater on campus than in the home; the factors associated with SHS exposure were location-specific.

## 1. Introduction

Secondhand smoke (SHS) is defined as the inhalation of tobacco smoke by nonsmokers against their will or as being involuntary exposure to tobacco smoke [1]. SHS consists of 15% mainstream smoke and 85% sidestream smoke. Mainstream smoke is the smoke discharged by expiration after being filtered through the smoker’s lungs, while sidestream smoke is the smoke that goes directly into the air from a burning cigarette or cigar. In particular, the sidestream smoke contains many highly concentrated toxic chemicals or carcinogens, which may be more harmful than mainstream smoke [2]. 

SHS exposure is associated with an increased risk of developing cancers and cardiovascular diseases. Studies have reported that nonsmokers with SHS exposure have a 2.1 times greater risk of developing lung cancer compared to those without SHS exposure [3], while they have a 1.6 and 1.4 times greater risk of developing cardiovascular disease [4] and stroke [5], respectively.

In Korea, the smoking rate of adult men over 19 years old was 67.6% in 2000, and 46.7% in 2009. However, the decreased smoking rate in 2008 is still high compared to the rates in other OECD (Organization for Economic Co-operation and Development) countries [6]. After the National Health Promotion Act was enacted in 1995, most public places have been designated as being no-smoking areas in Korea. Subsequently, the WHO Framework Convention on Tobacco Control, which came into force in 2005, has facilitated no-smoking policies of Korea [7]. As a result, a campaign for anti-secondhand smoke was launched in 2008, with the popular slogan, “Say No, Save Life”; this campaign aims to disseminate information regarding the harmful effects of SHS exposure and encourages people to be assertive of their dislike of SHS exposure [8]. However, despite the governmental efforts, it has been reported that 68% of nonsmoking Korean adults were exposed to SHS [9] and 31% of Korean children in the home [10]. 

College students may be vulnerable to unhealthy behaviors. Generally, a focus on academic achievement during the period of middle and high schools in Korea makes students passive toward their own healthy lifestyle habits. As entering universities, college students must for the first time make their own health decisions. However, studies have reported that college students have lower levels of health promoting behaviors than adults [11]. With regard to smoking behavior, current smoking rate stands of 32.4% for ages 19–29 (51.9% for men and 11.1% for women), which was much greater than 26.6% for the general population over 19 years old [12]. College students may be a representative population for ages 19–29 in Korea because attainment rates of university-level education in Korea are relatively high (63%) among OECD countries. These data indicate that college students are more prone to SHS exposure than the general population. College students spend most time per day on campus and in the home. However, few studies reported SHS exposure by location and its associated factors among college students.

Therefore, we aimed to determine the prevalence and frequency of SHS exposure among nonsmoking college students at two locations, *i.e.*, on campus and in the home, and to identify factors associated with SHS exposure at each location. The results from the study may yield insights into the current status of SHS exposure among college students and could provide a framework for the development of policy strategies for reducing smoking and SHS exposure on campus and in homes.

## 2. Methods

### 2.1. Design and Participants

A cross-sectional study was performed to identify factors associated with SHS exposure among college students in Korea, as a preliminary stage for a nationwide large-scale funded study. Participants were 2209 college students recruited by convenience sampling from a total population of 25,373 students of two universities in Seoul and Gyeonggi-do in Korea (38.7% of all university students in Korea attend universities located in Seoul and Gyeonggi-do). Of the 2209 college students, 1754 nonsmoking college students (79.4%) were included in the final analysis, after excluding 455 students (20.6%) who currently smoke. We conveniently selected about 10 departments at each university and approached instructors or teaching assistants to obtain approval for administering questionnaires in class. After gaining approval, trained research assistants visited these departments in person to explain the objectives of the present study during the period from September to December 2009. The research assistants distributed copies of the questionnaires and gave verbal instructions. Students who consented then completed the anonymous questionnaires by themselves during class breaks and returned them. In addition, we recruited volunteer students from a campus booth, who were willing to take time to respond to the questionnaire. The participation rate was approximately 75%. 

### 2.2. Measures

Two questions were asked about SHS exposure. The first was, “During the last month, have you been exposed to secondhand smoke on campus or in the home?” [13]. In this question, participants were additionally asked to specify the indoor places where they had experienced SHS exposure on campus, namely, in restrooms, student lounges, cafeterias, libraries, student clubs, lecture rooms, and/or campus stores. The second question was about the frequency of SHS exposure [13,14]: “If you were exposed to SHS in the last month, how many times per week were you exposed?” Other questions were about socio-demographic, health-related, and environmental characteristics that may influence SHS exposure [9]. For the health-related characteristics, current illness was defined as the presence of current medical problems, including asthma, heart diseases, cancers, hypertension, diabetes, and other serious diseases being treated. Self-rated health was queried by rating it as excellent, very good, good, fair, or poor, and then collapsed into the categories good (excellent, very good, and good) and poor (fair and poor). Family member’s illness was defined as the presence of current medical problems among any family members living with the participant at home. For environmental characteristics, exposure to anti-SHS information included any previous anti-SHS TV and radio campaigns, newspapers, or educational programs (Table 1). 

**Table 1:** Characteristics of the participants (n = 1754).

**Sociodemographic characteristics**		**n (%)**
Age (years)	17–19	722 (41.2)
	20–21	663 (37.8)
	≥21	369 (21.0)
Gender	Female	1323 (75.4)
	Male	431 (24.6)
Grade level	Freshmen	914 (52.1)
	Sophomores	559 (31.9)
	Juniors	211 (12.0)
	Seniors	70 (4.0)
Married	yes	33 (1.9)
**Health-related characteristics**		
Current illness	yes	120 (6.8)
Self-rated health	Good	1581 (90.1)
	Poor	173 (9.9)
Smoking status	Non-smoker	1638 (93.4)
	Ex-smoker	116 (6.6)
Family member’s illness	yes	610 (34.8)
**Environmental characteristics**		
Father’s education	Some college or more	883 (50.3)
Mother’s education	High school degree or less	1224 (69.8)
Family member smokes	yes	820 (46.8)
Friend smokes	yes	663 (37.8)
Exposure to anti-SHS information	yes	1274 (72.6)

SHS = secondhand smoke, SD = standard deviation

### 2.3. Statistical Analysis

The data were analyzed with SPSS 18.0 (SPSS, Inc., Chicago, IL, USA, 2008) and the significance level was set at 0.05. To examine the prevalence of SHS exposure by participants’ characteristics at each location, *i.e.*, on campus and in the home, a chi-square test was performed. To identify the factors associated with SHS exposure, a multiple logistic regression analysis was performed after placing predictor variables into regression models with the outcome variable of SHS exposure. These predictor variables were included in the regression models, primarily based on previous studies [9,10]; they were placed in the models by location, because potential correlates with SHS exposure by location may differ between campus and home. 

## 3. Results

The participants (n = 1754) had a mean age of 20.5 years; 75.4% were female students and 52.1% were freshmen (Table 1). In total, 6.8% had current illness and 9.9% rated their health as poor; 46.8% had a family member who smoked, 37.8% had a friend who smoked, and 72.6% had been exposed to anti-secondhand smoke information during the past month.

Of the total participants, 83.1% (n = 1458) were exposed to SHS at least once per week on campus or at home (Table 2). Specifically by location, 79.7% (n = 1398) were exposed to SHS on campus on average 3.0 times per week; of these, 80.5% were exposed to SHS in restrooms, followed by 26.7% in student lounges, 14.2% in cafeterias, 8.1% in libraries, 7.9% in student clubs, 7.7% in lecture rooms, and 6.2% in campus stores. Of the total participants, 23.5% (n = 412) were exposed at home on average 3.7 times per week. 

**Table 2:** Prevalence and frequency of secondhand smoke exposure (N = 1754).

	Exposure	Frequency of exposure ^a^
Location	n (%)	Mean (SD)
Overall (home or campus)	1458 (83.1)	3.4 (2.89)
Home	412 (23.5)	3.7 (3.24)
Campus	1398 (79.7)	3.0 (2.53)
Restrooms	1126 (80.5)	4.0 (2.98)
Student lounges	373 (26.7)	3.2 (2.73)
Cafeterias	198 (14.2)	2.5 (2.30)
Libraries	113 (8.1)	2.7 (2.72)
Student clubs	111 (7.9)	2.4 (2.63)
Lecture rooms	108 (7.7)	3.6 (2.12)
Campus stores	86 (6.2)	2.5 (2.22)

SD = standard deviation. ^a^ Frequency of exposure per week at each location was elicited from exposed students.

The SHS exposure on campus correlated significantly and inversely with grade levels (χ^2^ = 31.27, *p* < 0.001) (Table 3). SHS exposure in the home was significantly more prevalent among female students (χ^2^ = 7.72, *p* = 0.003), married students (χ^2^ = 4.73, *p* = 0.029), those who rated their health as poor (χ^2^ = 9.55, *p* = 0.002), those with less educated parents (defined as below college level education, χ^2^ = 13.04 for the father, *p* < 0.001, and χ^2^ = 7.35, *p* = 0.004, for the mother), and those with smokers in their families (χ^2^ = 292.05, *p* < 0.001).

**Table 3:** Secondhand smoke exposure by location (N = 1754).

Variables			Exposure on campus	Exposure in the home
		n	n (%)	n (%)
Age (years)	17-19	722	593 (82.1)	181 (25.1)
	20-21	663	525 (79.2)	150 (22.6)
	≥22	369	280 (75.9)	81 (22.0)
Gender	Female	1323	1058 (80.0)	332 (25.1) **
	Male	431	340 (78.9)	80 (18.6)
Grade level	Freshmen	914	743 (81.3) **	227 (24.8)
	Sophomores	559	463 (82.8)	131 (23.4)
	Juniors	211	150 (71.1)	43 (20.4)
	Seniors	70	42 (60.0)	11 (15.7)
Married	Yes	33	28 (84.8)	13 (39.4) *
	No	1721	1370 (79.6)	399 (23.2)
Smoking status	Ex-smoker	116	99 (85.3)	28 (24.1)
	Nonsmoker	1638	1299 (79.3)	384 (23.4)
Anti-SHS exposure information	Yes	1274	1027 (80.6)	302 (23.7)
	No	480	371 (77.3)	110 (22.9)
Self-rated health	Good	1581	1253 (79.3)	355 (22.5) **
	Poor	173	145 (83.8)	57 (32.9)
Current illness	Yes	120	97 (80.8)	31 (25.8)
	No	1634	1301 (79.6)	381 (23.3)
Friend smokes	Yes	1091	522 (78.8)	267 (24.5)
	No	663	876 (80.3)	145 (21.9)
Family member’s illness	Yes	610	-	150 (24.6)
	No	1144		262 (22.9)
Father’s education	Some college or more	883	-	239 (27.1) **
	High school degree or less	829		163 (19.7)
Mother’s education	<College	1224	-	308 (25.2) **
	≥College	489		93 (19.0)
Family member smokes	Yes	820	-	344 (42.0) **
	No	934		68 (7.3)

SHS = secondhand smoke; * *p* < 0.05, ** *p* < 0.01 for associations between characteristics and secondhand smoke exposure using chi-square tests.

Table 4 shows factors associated with SHS exposure based on the results of the multiple logistic regression analysis. On campus, freshmen and sophomores had a 2.99 and 3.42 times greater risk for SHS exposure, respectively, than seniors (*p* < 0.001 for all) (Table 4). At home, female students had a 1.49 times greater risk for SHS exposure than male students (*p* = 0.003). Those who rated their health as good had a 0.57 times lower risk for SHS exposure than those who rated their health as poor (*p* = 0.008). Those with smokers in their families had a 9.27 times greater risk for SHS exposure than those who did not (*p* < 0.001).

**Table 4:** Factors associated with secondhand smoke exposure by location (N = 1754).

		On campus		In the home	
Variables		Adjusted OR (95% CI) ^a^			
Age	17–19 20–21 ≥22	1.05 0.89 1.00	(0.69–1.59) (0.62–1.27)	1.25 1.15 1.00	(0.88–1.76) (0.81–1.63)
Gender	Female Male	1.22 1.00	(0.91–1.64)	1.49 1.00	(1.10–2.03) *
Grade level	Freshmen Sophomores Juniors Seniors	2.99 3.42 1.71 1.00	(1.61–5.57 ) * (1.89–6.18) * (0.95–3.10)	-	
Married	Yes No	1.79 1.00	(0.67–4.79)	2.18 1.00	(0.97–4.91)
Smoking status	Ex-smoker Nonsmoker	1.50 1.00	(0.87–2.58)	0.85 1.00	(0.51–1.40)
Anti-SHS exposure information	Yes No	1.29 1.00	(0.99–1.67)	1.29 1.00	(0.97–1.70)
Self-rated health	Good Poor	0.79 1.00	(0.51–1.21)	0.57 1.00	(0.38–0.85) *
Current illness	Yes No	0.95 1.00	(0.59–1.54)	0.91 1.00	(0.55–1.49)
Friend smokes	Yes No	1.07 1.00	(0.83–1.37)	-	
Family member's illness	Yes No	-		0.96 1.00	(0.74–1.24)
Father’s education	Some college or more High school degree or less	-		1.30 1.00	(0.97–1.76)
Mother’s education	Some college or more High school degree or less	-		0.90 1.00	(0.64–1.27)
Family member smokes	Yes No	-		9.27 1.00	(6.92–12.43) *

SHS = secondhand smoke, CI = confidence interval, OR = odds ratio; ^a^ Odds ratios adjusted for the studied variables in the model by location;* *p* < 0.01.

## 4. Discussion

The present study found that 83.1% of nonsmoking college students were exposed to SHS at two locations, *i.e.*, on campus or at home. Specifically, 79.7% and 23.5% were exposed to SHS on campus and in the home, respectively. On campus, SHS exposure was significantly more prevalent among freshmen and sophomores than among seniors. In the home, SHS exposure was significantly more prevalent among females, those with smokers in their families, and those who rated their health as poor. 

This is, to the best of our knowledge, the first study to explore SHS exposure on campus among college students. One study revealed that 65% of college students in the U.S. were exposed to SHS in restaurants or bars, but did not examine on-campus exposure [15]. We found that college students were more likely to be exposed to SHS on campus (79.7% of college students) than in other places. The SHS exposure on campus was much more prevalent than in the workplace. In Korea, 34.7% of nonsmokers ages 19 and over were exposed to SHS in the workplace [16]. The greater prevalence of SHS exposure on campus may indicate weaker implementation of anti-smoking policies on campuses in Korea. In the U.S., the American College Health Association has adopted a no-tobacco policy and encourages colleges and universities to be diligent in their efforts to achieve a 100% indoor and outdoor campus-wide tobacco-free environment [17]. In Korea, since smoking was banned in some public places in 1995 in accordance with the National Health Promotion Act of Korea, only some sections of campus buildings are designated as no-smoking areas, *i.e.*, lecture rooms, student lounges, campus cafeterias, and campus conference halls [18]. In our study, the indoor places in campus buildings where college students were exposed to SHS most frequently were restrooms (80.5%), followed by the student lounges, cafeterias, libraries, student clubs, lecture rooms, and campus stores. Based on the National Health Promotion Act of Korea, our findings indicate that such a campus smoking policy is weak, has been ignored on campuses, and is not followed by college students and administrators. Korea signed the WHO Framework Convention on Tobacco Control (FCTC) in 2003, which has been implemented in Korea since 2005. The FCTC includes obligations to protect the public from exposure to tobacco smoke under the Article 8 of the Convention. This stipulates that all indoor public places, all indoor workplaces, all public transport and other public places should be free from exposure to SHS. Indoor places in campus buildings may be considered as indoor public places, based on the FCTC definition of public places, covering all places accessible to the general public or places for collective uses. Thus, the National Health Promotion Act of Korea should be extended to cover locations in campus buildings that are not included in the current version of the law, namely, those areas which this study found to be prevalent indoor places for SHS exposure–restrooms, libraries, student clubs and campus stores. The designation of smoking-free areas and public notices of such zones may influence smoking behavior of smokers. Sim *et al.* has reported that 69.2% of smokers would never smoke at public places under no-smoking sign boards [19]. Apel *et al.* reported that the designation of smoking-free areas in campus buildings resulted in a decrease in the number of cigarettes smoked [20]. Borders *et al.* found that clear identification or designation of non-smoking areas on campus significantly decreased the odds of smoking by 45% [21]. However, Warren *et al.* reported that 61.7% of college students had not seen no-smoking signs on their campus [22]. Hence, college administers should clearly designate smoking-free areas on campus so that college students may be aware of the presence of designated smoking-free areas. Such a policy in campus buildings should be closely monitored and modified by expanding it to100% smoking-free campuses. 

The present findings showed that freshmen and sophomores were exposed to SHS on campus more frequently than seniors. This observation may be explained by increasing population of smokers in the transition period from school-aged children to early young adults. The Korean Juvenile Protection Act prohibits tobacco selling to school-aged children under 19 years [18]. Students are almost 19 years old when they start college in Korea. For this reason, freshmen and sophomores can not only begin to purchase cigarettes without any restrictions, but also smoke freely in front of others without consideration. The smoking rate was reportedly 18.1% among male high school students, which increased sharply to 51.9% among male adults aged 20–29 years [12,18]. In this context, smoking preventive education may be a strategy for nonsmoking freshmen not to begin smoking or for college students who were not regular smokers in high school not to smoke. Preventive education programs held on campus were reported to decrease smoking rates by 33% to 30% [21]. Such programs may be included as a routine orientation session for freshmen before starting regular classes. 

In our findings, of nonsmoking college students, 23.5% were exposed to SHS at home. These findings were similar to those in a previous study. Hughes *et al.* reported that the prevalence of SHS exposure in the home was 29% among the age group of 18–35 years [10]. In addition, our findings showed that SHS exposure in the home was 9.27 times greater for college students who had smokers in their families than those who did not. Such a positive association between SHS exposure and family member’s smoking at home has been reported in women but not in men [9]. Similarly we also found the association only in women but not men (data not shown). In women, the SHS exposure associated with the existence of smokers in their families may be, in part, attributable to patriarchal power relations between men and women, which are embedded in the Korean traditional culture. For Korean men, smoking is a means for facilitating social relationships and a symbol of representing male identity [23]. Altogether, SHS exposure in the home may occur potentially under several circumferences, for example, when the spouse smoked, when the parents smoked, when smoking was allowed in the home, and when fewer groups discouraged smoking at home [10]. Therefore, policies and programs to discourage smoking at home would be needed. 

This study has some limitations. First, the participants of the present study may not be quite representative of the general population of Korean college students because they were recruited by convenience sampling. Given that the participation rate was approximately 75%, and 75.4% of the participants were female, a selection bias cannot be excluded in the present study. However, this study was, to the best of our knowledge, the first to address SHS exposure and its associated factors on campus among college students. The data were required as a preliminary basis for conducting a large-scale funded study in the future. In this context, when formulating a large-scale study, we need to reduce selection bias. Second, SHS exposure was retrospectively assessed by self-report, which may produce recall bias. Thus, in a later study, the combination of a biomarker, e.g., urinary cotinine or hair nicotine levels, with self-reports of SHS exposure may be more accurate in assessing exposure to SHS [24,25], and in addition, provide information on how the self-reported levels of SHS exposure correlate with levels of such biomarkers.

## 5. Conclusions

SHS exposure was common among nonsmoking college students at two universities in Korea, with more than two-thirds exposed on campus or in the home. The prevalence of SHS exposure was greater on campus than in the home. On campus, SHS exposure was significantly more prevalent in freshmen and sophomore students. In the home, SHS exposure was significantly more prevalent among females, those with smokers in their families, and those who rated their health as poor. Therefore, location-specific strategies, for example, stronger regulation and monitoring of designated smoking-free areas on campus or preventive smoking programs for freshmen, should be developed for protecting nonsmoking college students from SHS exposure. 
